# Stability research on polydopamine and immobilized albumin on 316L stainless steel

**DOI:** 10.1093/rb/rbw030

**Published:** 2016-09-20

**Authors:** Hao Zhang, Lingxia Xie, Jinchuan Deng, Weihua Zhuang, Rifang Luo, Jin Wang, Nan Huang, Yunbing Wang

**Affiliations:** ^1^School of Material Science and Engineering, Southwest Jiaotong University, Chengdu, 610031, China;; ^2^National Engineering Research Center for Biomaterials, Sichuan University, Chengdu, 610064, China

**Keywords:** polydopamine, thermal, stability, X-ray photoelectron spectroscopy (XPS), catechol

## Abstract

In this study, the polydopamine (PDA) film was coated on polished 316Lss and then thermally treated at 150 °C (labeled as PDA-Th150), and the stability of coatings was also investigated. Straining test indicated that PDA-Th150 coating performed better in affording sufficient adherence to 316 L SS substrate. Moreover, both PDA and PDA-Th150 coating suffered slight swelling during immersion in deionized water (pH = 6.5). X-ray photoelectron spectroscopy results showed that during immersion, latent nucleophilic reaction via amines inside PDA coating occurred. This led to an enhanced cross-linking and thus gradually promoted the coating stability. Moreover, larger amount of bovine serum albumin (BSA) was immobilized onto PDA-Th150 coating and performed well in anti-platelet adhesion. A high retention of immobilized BSA was observed even after immersion for 30 days. These tests suggested that PDA was stable enough and performed well in surface functionalization, which might enrich the research and application of PDA.

## Introduction

Surface properties are considered to be of significance and surface modiﬁcation techniques are highlighted in current material design for obtaining desirable effects [1–3]. In relation to biomedical research and applications, many efforts have been devoted to design engineer surface for desirable properties, including bioactive coatings, biomimetic construction and plasma treatment [4–7]. Biomolecule immobilization is a facile and effective approach. However, most of current materials need an ad-layer/platform for the further implement of biomolecule immobilization [8, 9].

Inspired by the adhesive proteins secreted by marine mussels, polydopamine (PDA) was wildly investigated to implement surface modiﬁcation for both inorganic and organic materials [10, 11], bridging the gap between hydrophobic/hydrophilic and bio-inert/bio-active materials conveniently. Facing the potential of PDA in biomedical research area, Kang *et al.* [12] reported a biofunctionalization scheme for neural interfaces using PDA polymer. Lynge *et al.* [13] found that surface modification of PDA coating could improve the adherence and proliferation of myoblast cells. Additionally, Hong *et al.* [14] found that PDA functionalized surface also declined the toxicity *in vivo*. Moreover, series of biomolecules have been immobilized onto PDA surface and introduced desirable effects [15–19]. These results indicated that PDA was an effective way in surface functionalization.

Generally speaking, the high adhesion strength of the coatings was crucial to fabricate durable devices [20]. Considering the potential of PDA applied in medical devices, the adherent performance of coating and metal substrate is important. Hereon, the straining test was adopted to help evidence the coating adhesion properties on 316 L SS. Note that, the stability of such coatings is crucial for long-term service. 316 L stainless steel (316 L SS) was used as the substrate because this kind of material has already been widely demonstrated for its application in clinic, such as vascular stent. 316 L SS is a commonly used stent material because of its suitable mechanical properties and excellent corrosion resistance. Besides, 316 L SS is truly a proper substrate for the investigation of coating ability of PDA PDA and the corresponding mechanical properties. In this work, the thermal performance and coating stability of PDA on 316 L SS substrate were investigated. Moreover, the evaluation of the stability and effectivity of immobilized bovine serum albumin (BSA) were also performed to evidence this platform. This work also aimed at supplementing the research and application of PDA.

## Experimental

### Materials

316 L SS was obtained from New Material Co., Ltd (Xi’an, China). Dopamine, Tris (hydroxymethyl) aminomethane (Tris-base), BSA (MW 68 000) were purchased from Sigma-Aldrich (USA).

### Preparation of PDA coating

The mirror polished 316 L SS (Φ = 10 mm) were deposited in 2 mg/ml dopamine solution (10 mM Tris, pH = 8.5) at 20°C for 12 h. After that, PDA coated 316 L SS were ultrasonically washed in distilled water (10 min, three times). The PDA coatings were subsequently thermally treated at 150°C for 1 h and designed as PDA-Th150.

### Characterization

The surface chemical composition was investigated via X-ray photoelectron spectroscopy (XPS, Perkin-Elmer 16PC) with a monochromatic Al K α excitation radiation (hν = 1486.6 eV). The containment carbon (C1s = 284.7 eV) was used as calibration. The surface roughness was observed by atomic force microscopy (AFM, SPI 3800, NSK Inc., Japan). The static drops by using DSA100 (Krüss, Hamburg, Germany) were tested to analyze the contact angles of samples. The method was depicted by the manufacturer at 25°C and 60% relative humidity.

### Straining test

The substrate straining method was used to determine the mechanical properties of coatings [21, 22]. Based on the study of Zhang *et al.* [23], a model was developed. Using an Instron Tensile tester machine, samples were loaded in tension at a constant crosshead velocity of 0.5 mm/min to the desired strain state. Substrates stretch elongations rates were strained to 40 and 80%, respectively (Hereafter labeled as PDA-8 mm, PDA-16 mm, PDA-Th150-8 mm and PDA-Th150-16 mm). Scanning Electron Microscope (SEM, Quanta 200, FEI, Holland) was used to study the development of cracks in PDA and PDA-Th150 coated 316 L SS substrate.

### BSA immobilization

The BSA was immobilized by incubation of the PDA and PDA-Th150 coated 316 L SS in a solution of 2 mg/ml BSA (10 mM deoxygenated Tris buffer, pH = 8.5) at room temperature for about 12 h. The substrates were respectively signed as PDA-BSA and PDA-Th150-BSA. To monitor the immobilization ability, Quartz Crystal Microbalance with Dissipation Monitoring (QCM-D) technique was adopted, which is a good candidate for evaluation of surface-related processes in liquids [24]. In brief, coatings were deposited on the AT-cut 5 MHz Au coated quartz crystal (diameter of Au films: 10 mm) and then exposed to PBS (pH = 7.3) fluid at 50 μl/min to remove unstable PDA. Actually after coating for 12 h, the substrate are fully covered with PDA components (usually the thickness is larger than 15 nm). Thus we speculate that after the fully covering of PDA components, it did not show too much difference in addressing the research on the secondary reactivity of PDA. So coating Au substrate and testing the biomolecule immobilization ability on PDA coating is reasonable. The 2 mg/ml BSA solution (2 mg/ml, Tris, pH 8.5) was passed through the chamber in contact with the crystal. The frequency shift (Δf) was related to the adsorbed mass (Δm) according to the Sauerbrey relation [25].

(1) Δm=Δ f×C/ n

*C* (*C* = 17.7 ng/cm^2^ HZ^−1^ at *f*_n_ =  5 MHz) is the mass-sensitivity constant and *n* (*n* = 1, 3, 5, …) is the overtone number.

### Stability in deionized water

Water ageing assessment is considered as the first test to evaluate the stability of coatings and immobilized biomolecules on 316 L SS substrate [26]. Static stability tests of PDA coatings and immobilized BSA were carried out at 37°C for a period ranging from 1 to 30 days in deionized (D.I.) water (pH = 6.5). The chemical and physical properties of the coatings were detected by different surface analysis techniques like XPS and SEM.

### Platelet adhesion test

Fresh human whole blood was legally obtained from the central blood station of Chengdu, China in agreement with ethical standards. And the experiments were approved by the Institutional Review Board in Chengdu Medical College. The whole blood was anti-coagulated with tri-sodium citrate in a 9:1 volumetric ratio. Fresh human blood was centrifuged at 1500 rpm for 15 min to obtain the top layer of platelet rich plasma (PRP). After that, 60 μl of PRP was added onto the sample surfaces placed in a 24-well plate and then incubated for 2 h at 37°C. Then the samples were carefully rinsed with PBS to remove non-stable platelets. The samples were washed with D.I. water for three times after fixed with 2.5 wt% glutaraldehyde for 12 h. The platelets adsorbed on the surfaces were dehydrated with 30, 40, 50, 70, 90 and 100 vol% ethanol/water solution for 15 min for each successively. The morphologies of platelets on resultant samples were examined by SEM.

## Results and discussion

### Surface chemistry, morphology and wettability

Previous studies indicated that thermal treated PDA presented increased quinone and correspondingly decreased amine and catechol content [27]. Briefly, catechols were oxidized to quinones and primary amino groups were somewhat consumed after thermal treatment. PDA-Th150 coating was less hydrophilic with a water contact angle (WCA) value of 86.4° compared with PDA coating (66.3°). What’s more, PDA and PDA-Th150 surfaces contained 33.5 and 48.2% ratio of quinones respectively, indicating PDA-Th150 coating was more effective when served as a biomolecule immobilization platform.

As a widely applied ad-layer, the practical performance of PDA was considered to be of significance. Considering the potential of PDA-Th150 when served as a platform for biomolecule immobilization, detailed evaluations on mechanical properties and stability were urgent. As shown in Supplementary Figure S1 and Supplementary Table S1, after deposition and thermal oxidation, both PDA and PDA-Th150 surface presented larger root mean square (RMS) roughness than 316 L SS. Ratner *et al.* [29] demanded that, the surfaces of blood contacting materials like cardiovascular stents should be smooth with the roughness at the level of protein adsorption (<50 nm), otherwise platelets adhesion and thrombogenesis may occur. Thus, the PDA and PDA-Th150 modified 316 L SS is low enough at surface roughness aspect for safe hemocompatibility. Interestingly, after thermal treatment of PDA at 150°C, a much smoother ﬁlm emerged with the RMS roughness of 10.8 ± 2.3 nm, lower than 16.8 ± 4.9 nm of PDA. The mean grain size and ratio of grain area were also lower on PDA-Th150 surfaces. As investigated by Wei *et al.* [31] , unreacted dopamine monomers or PDA incorporated into the coating by noncovalent bond were existed after polymerization process. Thus, after thermal treatment, these unstable contributions were released from the coating. Besides, the smoother surface should enable a more uniform interfacial contact between coatings and substrates [30].

**Figure 1.**
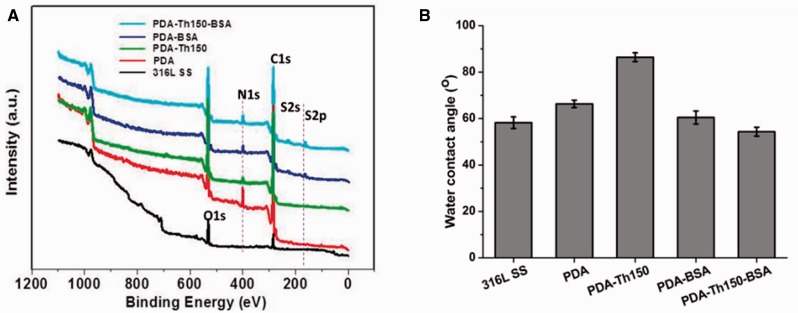
(A) XPS wide scans for the unmodiﬁed and different modified 316 L SS membranes; **(B)** the WCA of different samples

A series of investigations have been carried out on Schiff base and Michael addition reactions of PDA [11]. Figure 1A showed the XPS wide scan of different samples. The appearance of sulphur signal indicated that BSA was successfully immobilized onto PDA and PDA-Th150 coating surfaces. After BSA functionalization, the surfaces became more hydrophilic due to the introduction of the more hydrophilic groups (–NH_2_ and –COOH) in BSA (Fig. 1B). These are in good agreement with published results [19, 31].

### Straining test

**Figure 2.**
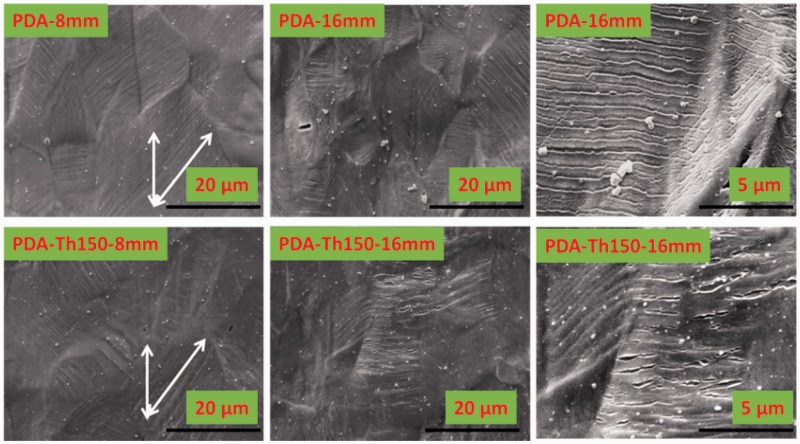
SEM Results after tensile deformation of PDA and PDA-Th150 modified 316 L SS (bar: 20 μm in 3000× and 5 μm in 10 000× magnification)

Because PDA may be served on substrates of different species and sizes, and even may suffer bending or stretching treatment. The integrity of PDA coatings was a key demand for maintaining the safety of coatings. The performance may be quite different considering the different adhesion mechanism on different material substrates like polymers and metals. Yang *et al.* [32, 33] coated PDA on polydimethylsiloxane (PDMS) and found that it was able to coat plastic, ceramic and metal surfaces and join or bond rigid substrates but might not be suitable for joining soft or flexible parts. Figure 2 displayed the tensile deformation of PDA and PDA-Th150 coated 316 L SS at different strain state. When the stretch elongation rate of substrate was implemented at 40%, most of the striated textures were aligned at a direction of ±45° to the tensile axis, suggesting good adherence between coating and stainless steel substrate. During the strain of the samples, the coatings and substrates were strained integrally, if the coatings kept the movement similar to the substrate first and then detached from substrates, the strained cracks would show a direction of ±45° to the tensile axis, suggesting good adhesion strength [34]. Otherwise, the coatings will detach from substrates directly in the early stretching without a direction of ±45° of cracks. Both PDA and PDA-Th150 coating presented the accompanying displacement with 316 L SS, yet still adhered to the substrate with no obvious cracks. Although the elongation increased to 16 mm, interfacial debonding and cracks occurred between PDA and substrate. Local cracks even were lifted and divorced from the substrate. However as to PDA-Th150 coating, though cracks emerged, they were more isolated and didn’t form the overall stripping, suggesting better adherence to the substrate even after this violent straining. Base on above analysis, the smoother surface should enable a more uniform interfacial contact between PDA-Th150 and stainless steel. This tensile deformation test, in another aspect, also evidenced the phenomenon in our stent expansion evaluation. Thermal performance of PDA revealed a more stable and cohesive property with modified 316 L SS substrates.

### Stability in D.I. water

As commonly accepted, good stability is essential to ensure the security of implanted devices *in vivo*. As a polymer coating, three main processes will usually happen during aqueous immersion: swelling, dissolution and hydrolysis. Static stability tests of PDA coatings were carried out at 37°C for a period ranging from 1 to 30 days in D.I. water (pH = 6.5). Unlike PBS or Hank’s solution, D.I. water was adopted so that we could follow the composition information without any contaminations coming from ion adsorption when pseudo physiological media were used.

**Figure 3.**
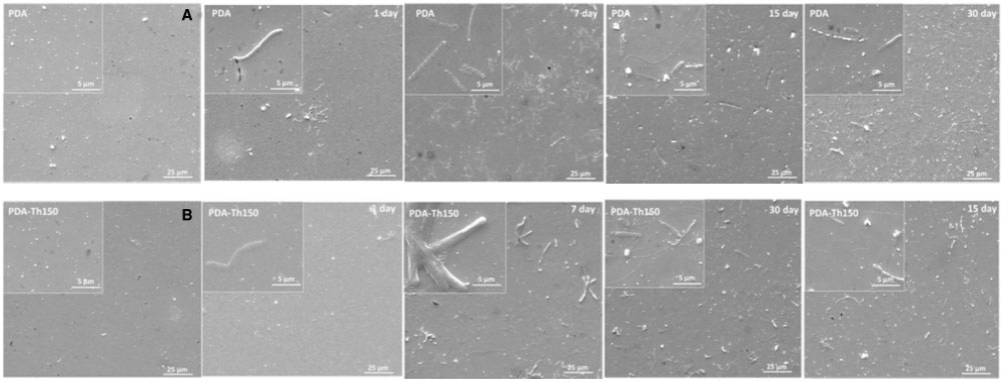
SEM images of the PDA and PDA-Th150 coating modified 316 L SS before and after immersing in D.I. water for various periods of time

As shown in Figure 3, the coatings were homogeneous and continuous before immersion and a slight swelling emerged after immersion of 1 day. During immersion, only the swelling occurred, indicating good stability of PDA coatings. As accepted, under a normal swelling process, water will penetrate into the polymer coating and gradually lead to an increased swelling degree during immersion. Interestingly, it seemed that among the observation of swelling at four time points, coatings immersed for 7 days presented the most serious swelling degree. In consideration of not only the size of swelling stripe, but also the total swelling area, enhanced anti-swelling phenomenon was found on both PDA and PAD-Th150 coating after immersion for 15 and 30 days. Based on this, we wondered that whether there were latent reactions inside the PDA coating during immersion.

**Figure 4.**
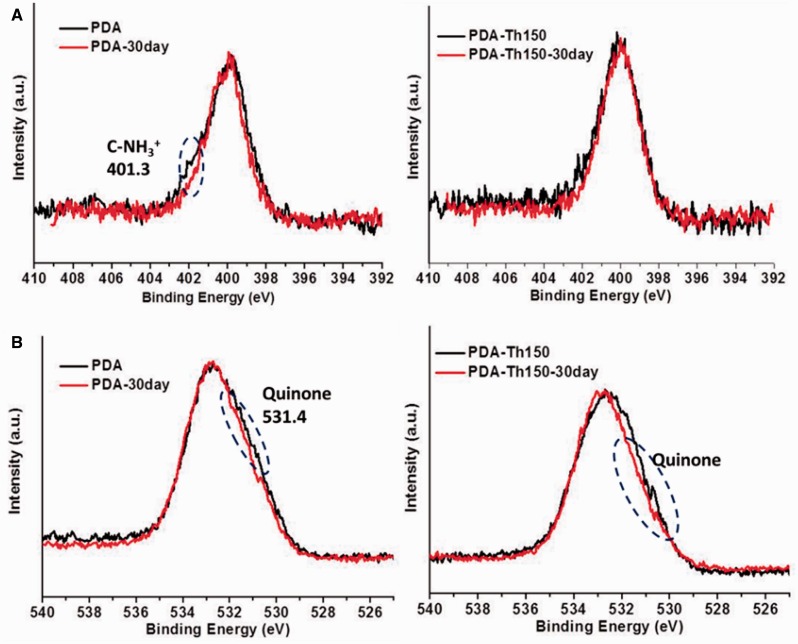
XPS spectra of N1s **(A)** and O1s **(B)** of PDA and PDA-Th150 before and after immersion in D.I. water for 30 days

**Figure 5.**
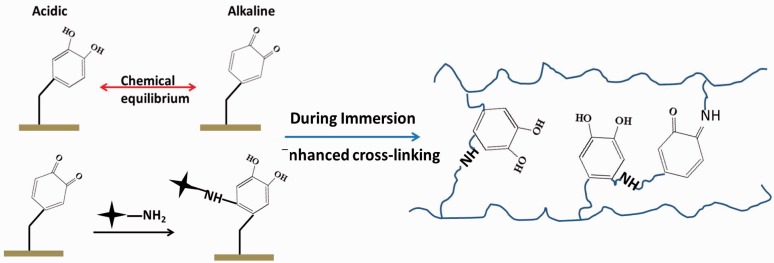
Illustration of the chemical equilibrium between catechol and quinone and the potential intramolecular reaction, and enhanced cross-linking degree and anti-swelling property during immersion in D.I. water

Complex redox reaction existed in dopamine polymerization and after deposition, there existed catechols, quinones and primary amino groups inside the coatings. These functional groups still had reactivity when placed in a proper aqueous condition. Using XPS technique, we surveyed the detailed information to better understand what happened during immersion in D.I. water. Lee *et al.* [11, 35] reported that, at a lower pH value, chemical equilibrium of NH_2_/${\text{NH}}_{3}^{+}$ would shift toward NH_3_^+^ species. In our immersion test, the amines on PDA, at acidic pH, were protonated. Based on the chemical equilibrium of NH_2_/${\text{NH}}_{3}^{+}$, it was believed that the content of amines would also reflect the potential content of protonated C-NH_3_^+^ species. According to Figure 4, obvious chemical shift occurred indicating that the ratio of chemical binding style in PDA coating changed. As shown in Figure 4A, the contribution around 401.3 was ascribed to NH_3_^+^. After immersion, the peak stood for NH_3_^+^ species dramatically weakened in PDA. This implied that the content of amine groups on PDA also decreased during immersion. Moreover, catechol/quinone states were in equilibrium in aqueous media, with the equilibrium shifted toward the quinone at alkaline pH [11]. According to Figure 4B, quinones on both PDA and PAD-Th150 coatings shifted obviously to catechols after immersion for 30 days, demonstrating that catechol/quinone equilibrium would possibly shift toward catechol at acidic pH (Fig. 5). Based on above data and Lee’s job, PDA was believed to react toward amines at a pH 5.5–9.5 aqueous solution. We speculated that during immersion, seen in Fig. 5, the amines would perform like nucleophiles and could undergo a 1, 4 Michael addition. The consuming of amines would also induce the decrease of ${\text{NH}}_{3}^{+}$ species after protonation. Though existed, the chemical shift of ${\text{NH}}_{3}^{+}$ was found less obvious on PDA-Th150 coating before and after immersion because the initial content of primary amino group content was lower on PDA-Th150 surfaces due to the thermal oxidation process [27]. Noteworthily, the reaction via amines would partially lead to an enhanced cross-linking inside PDA coating. Polymers containing higher cross-linking density will present better anti-swelling properties [36, 37]. Thus, the enhanced cross-linking density will help PDA perform well in anti-swelling. This investigation provided a primary explanation for the mild swelling of both PDA and PDA-Th150 after immersion of 15 and 30 days.

**Figure 6.**
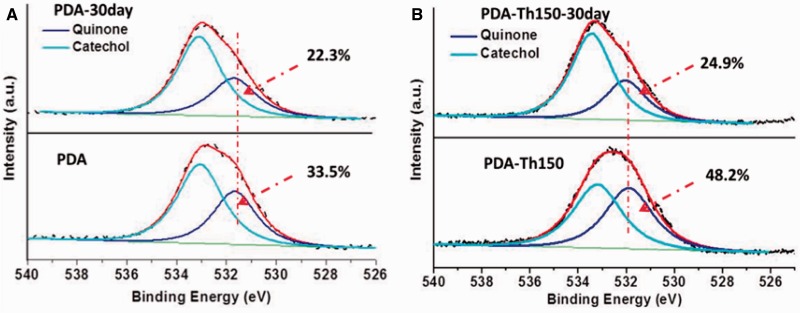
Curve ﬁtting results of O1s of PDA and PDA-Th150 coating before and after immersion in D.I. water for 30 days

Additionally, detailed curve fitting results of O1s were exhibited in Fig. 6 and Supplementary Table S2 and S3, after immersion for 30 days the quinone content decreased from 33.5 to 22.3% and 48.2 to 24.9% on PDA and PDA-Th150 surfaces, respectively. Note that, the latent reactivity toward nucleophiles was also a function of catechol/quinone chemical equilibrium [11]. Thus, not only the acidic pH caused the equilibrium toward catechol, but also the nucleophilic addition would make the contribution to the increased catechol content.

### BSA immobilization and platelet adhesion

**Figure 7.**
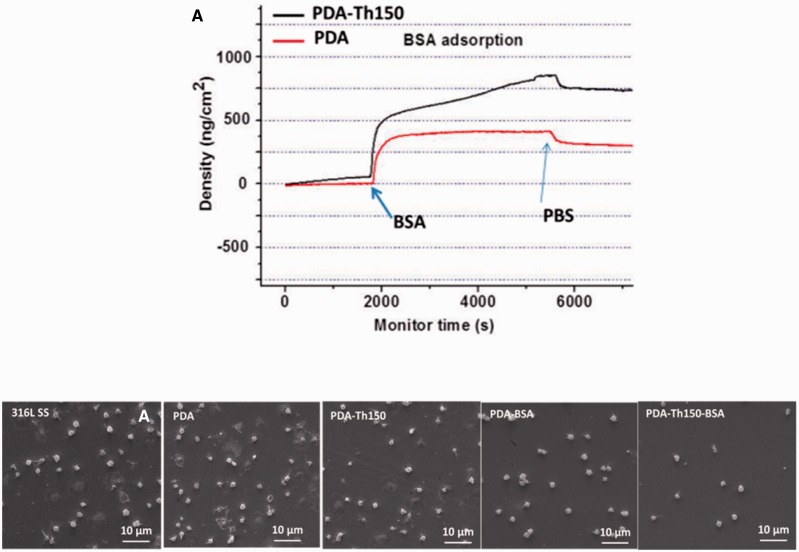
(A) QCM-D real-time monitoring for BSA immobilization on PDA and PDA-Th150 modified surfaces; **(B)** morphology of platelet adhesion results on different samples (2 h incubation in PRP) observed by SEM

In previous study, we demonstrated that quinone-rich PDA was more effective in biomolecule immobilization. Water soluble protein BSA was chosen and the platelet adhesion test was carried out to evaluate the effect of immobilized biomolecule. Figure 7A showed the QCM-D measurement of BSA immobilization. PDA-Th150 was also more effective than PDA in BSA immobilization (680 ng/cm^2^ on PDA-Th150 surface, compared with 300 ng/cm^2^ on PDA surface). Table 1 presented the elemental percentages on different samples obtained by XPS analysis. The S ratio was 0.8% on PDA-Th150-BSA surface, higher than that on PDA-BSA surface (0.5%), indicating the good agreement with QCM-D analysis and WCA results.

**Table 1.** Surface elemental percentages of PDA, PDA-Th150, PDA-BSA and PDA-Th150-BSA membranes (data obtained via XPS analysis)

Sample	C (%)	*n* (%)	O (%)	S (%)
PDA	76.6	7.5	15.9	
PDA-Th150	75.0	5.9	19.1	
PDA-BSA	70.0	10.7	18.8	0.5
PDA-Th150-BSA	66.8	11.7	20.7	0.8

As can be seen in Fig. 7B, compared with unmodified PDA, PDA-BSA and PDA-Th150-BSA inhibited the platelet adhesion properties significantly. The BSA-immobilized samples could decrease the adhesion and activation of platelets. These results showed that the PDA modified 316 L SS reduce the rate of platelet adhesion. Moreover, the PDA-Th150-BSA surface showed the lowest amounts of adhered platelets on the PDA-Th150-BSA surface (Supplementary Fig. S2). The effect was also associated with the immobilized BSA content. Additionally, BSA modified samples were also immersed in D.I. water for different periods of time. The retention of S (%) suggested that though after 30 days’ immersion, immobilized BSA was stable on PDA surface (Supplementary Table S4). Thanks to the good stability of both PDA and PDA-Th150 surface, immobilized biomolecules could stand stable, evidencing the safety of PDA platform.

## Conclusion

In this work, PDA modified 316 L SS was thermally treated at 150°C (PDA-Th150), and the thermal performance as well as the stability of PDA coating were investigated. It was found that, after straining, thermal coating performed better in affording sufficient adherence to 316 L SS substrate. Moreover, there might exist latent cross-linking process when immerse both PDA and PDA-Th150 coating in D.I. water, which gradually promoted the coating stability. Due to the stability of PDA coating, the immobilized molecule (e.g. BSA) could stay stable and provide a high retention even after 30 days immersion. These thermal and stability tests suggested that PDA-Th150 was stable enough and performed well in surface functionalization and might enrich the research and application of PDA.

## Funding

This work is supported by the NSFC (Project 51173149), the Ministry of Science and Technology Project of China (Key Basic Research Project No. 2011CB606204).

*Conflict of interest statement*. None declared.

## Supplementary data

Supplementary data is available at *REGBIO* online.

Supplementary Figure S1
