# Supplementary material for: Stability research on polydopamine and immobilized albumin on 316L stainless steel
Source: Regen Biomater. 2016 Sep 20;3(5):277–84. doi: 10.1093/rb/rbw030 (PMC5043154; doi:10.1093/rb/rbw030)
Supplement: Supplementary Figure S1 [file Supplementary_material.docx]

***Supplementary Materials***

Stability research on polydopamine and immobilized albumin on 316L stainless steel

Hao Zhang^a^, Lingxia Xie^a^, Jinchuan Deng^a^, Weihua Zhuang^b^, Rifang Luo^b^*, Jin Wang^a^*, Nan Huang^a^, YunbingWang^b^*

a School of Material Science and Engineering, Southwest Jiaotong University, Chengdu 610031, China

b National Engineering Research Center for Biomaterials, Sichuan University, Chengdu 610064, China

*Corresponding author：Tel：+86 28 87634148；Fax：+86 28 87600625

E-mail address：[lrifang@126.com](mailto:lrifang@126.com) (R. Luo)；[jinxxwang@263.net](mailto:jinxxwang@263.net) (J. Wang)；[yunbing.wang@qq.com](mailto:yunbing.wang@qq.com) (Y. Wang)

**Contents**

Table S1 to S4

Figure S1 to S3

Table S1

Grain size information of PDA and PDA-Th150 coatings (data obtained via AFM analysis).

| Sample | Mean size (nm^2^) | Ratio of grain area (%) |
| --- | --- | --- |
| PDA | 7.9×10^4^ | 1.1 |
| PDA-Th150 | 5.5×10^4^ | 0.2 |

Table S2

Relative quinone and catechol content of PDA after different periods of immersion in D.I. water, obtained via curve fitting of O1s.

| Samples | 0 day | 1 day | 7 day | 15 day | 30 day |
| --- | --- | --- | --- | --- | --- |
| Catechol (%) | 66.5 | 67.9 | 71.4 | 75.9 | 77.7 |
| Quinone (%) | 33.5 | 32.1 | 28.6 | 24.1 | 22.3 |

Table S3

Relative quinone and catechol content of PDA-Th150 after different periods of immersion in D.I. water, obtained via curve fitting of O1s.

| Samples | 0 day | 1 day | 7 day | 15 day | 30 day |
| --- | --- | --- | --- | --- | --- |
| Catechol (%) | 51.8 | 57.0 | 64.8 | 70.9 | 75.1 |
| Quinone (%) | 48.2 | 43.0 | 35.2 | 29.1 | 24.9 |

Table S4

Surface elemental percentages of PDA-BSA and PDA-Th150-BSA membranes after immersion for 30 days (data obtained via XPS analysis).

| Sample | C (%) | N (%) | O (%) | S (%) |
| --- | --- | --- | --- | --- |
| PDA-BSA-30day | 69.2 | 10.9 | 19.5 | 0.4 |
| PDA-Th150-BSA-30day | 65.9 | 11.9 | 21.6 | 0.6 |


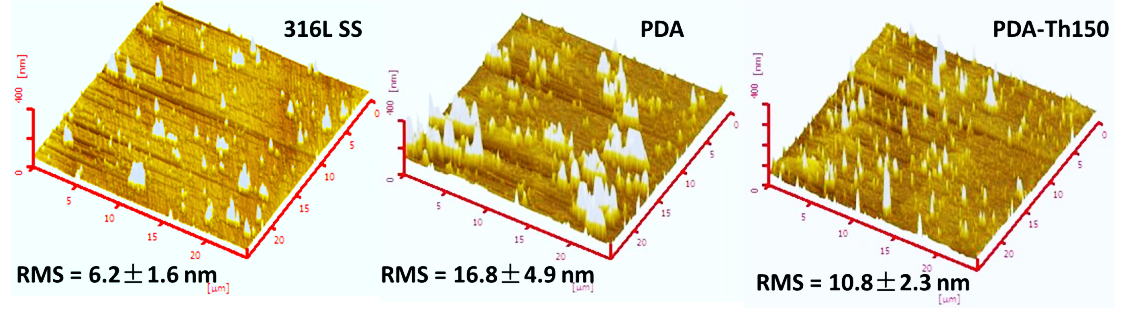


**Figure S1.** AFM tapping mode images of the 316L SS, PDA and PDA-Th150 modified 316L SS substrate.


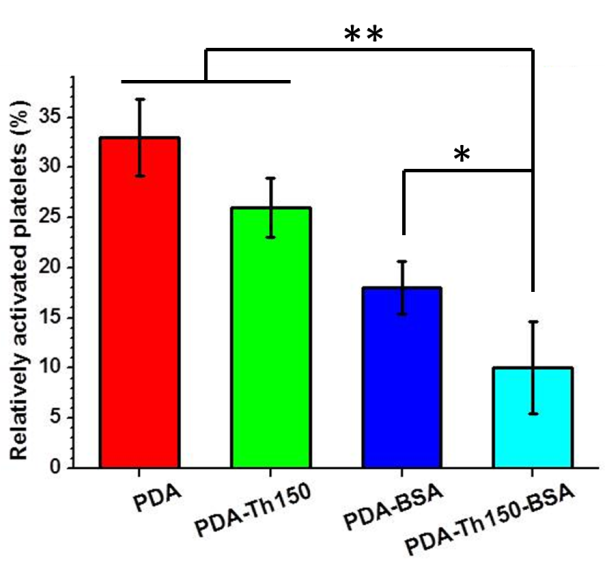


**Figure S2.** The relative quantification of activated platelets obtained by ELISA assays. Data expressed as mean ± SD (﹡p＜0.05, ﹡﹡p＜0.01)

*(Notes: This was done via assay of p-selectin:* Briefly, 60 μL of PRP was added onto each sample surface and incubated at 37 ° C for 2 h. After being rinsed with PBS (pH = 7.5, 5 min × 3 times), 20 μL of anti-CD62p (GMP-140, MCA796GA, Serotec Co.) dilution at a ﬁ nal ratio of 1:100 was added onto each sample and incubated for 1 h at 37 ° C. With PBS washed (5 min × 3 times), all the samples were incubated with 20 μL of horseradish peroxidase conjugated sheep anti mouse polyclonal antibody (second antibody, HRP, Catalog No: 074-1806, KPL Co.) at a dilution of 1:200 for 1 h at 37 ° C. After washed with PBS (5 min × 3times), 100 μL of 3,3′,5,5′-tetramethylbenzidine (TMB) chromogenic solution was added to react with the second antibody for 10 min. By adding 50 μL of H_2_SO_4_ (1M), the reaction was stopped. And the optical density was examined using a microplate reader at the absorbance of 540 nm.)
